# Ensemble Classifier for Epileptic Seizure Detection for Imperfect EEG Data

**DOI:** 10.1155/2015/945689

**Published:** 2015-02-04

**Authors:** Khalid Abualsaud, Massudi Mahmuddin, Mohammad Saleh, Amr Mohamed

**Affiliations:** ^1^Department of Computer Science & Engineering, College of Engineering, Qatar University, P.O. Box 2713, Doha, Qatar; ^2^Computer Science Department, Graduate School of Computing, University Utara Malaysia (UUM), 06010 Sintok, Kedah, Malaysia

## Abstract

Brain status information is captured by physiological electroencephalogram (EEG) signals, which are extensively used to study different brain activities. This study investigates the use of a new ensemble classifier to detect an epileptic seizure from compressed and noisy EEG signals. This noise-aware signal combination (NSC) ensemble classifier combines four classification models based on their individual performance. The main objective of the proposed classifier is to enhance the classification accuracy in the presence of noisy and incomplete information while preserving a reasonable amount of complexity. The experimental results show the effectiveness of the NSC technique, which yields higher accuracies of 90% for noiseless data compared with 85%, 85.9%, and 89.5% in other experiments. The accuracy for the proposed method is 80% when SNR = 1 dB, 84% when SNR = 5 dB, and 88% when SNR = 10 dB, while the compression ratio (CR) is 85.35% for all of the datasets mentioned.

## 1. Introduction

Brain status information is captured by physiological electroencephalogram (EEG) signals, which are extensively used to study different brain activities. In particular, they provide important information pertaining to epileptic seizure disease, as reported previously [1–3]. Epilepsy is a neurological disorder involving disturbances to the nervous system that are induced by brain damage. It has been reported [4] that 1% of the population worldwide is affected by this disease. Visual inspection of EEG signals can be very difficult and time consuming due to the difficulty of maintaining a high level of concentration during a lengthy inspection; this difficulty increases operator errors [5, 6]. Therefore, artificial intelligence techniques are proposed to enhance the process of epileptic seizure detection.

Recently, ensemble methods for EEG signal classification have attracted growing attention from both academia and industry. Sun et al. [7] evaluated the performance of three popular ensemble methods, namely, bagging, boosting, and random subspace ensembles. They reported that the capability of the ensemble methods is subject to the type of base classifiers, particularly the settings and parameters used for each individual classifier. Dehuri et al. [8] presented the ensemble of radial basis function neural networks (RBFNs) method to identify epileptic seizures. This method was based on the bagging approach and used differential evolution- (DE-) RBFNs as the base classifier. He et al. [9] proposed a signal-strength-based combining (SSC) method to support decision making in EEG classification. The results show that the proposed SSC method is competitive with the existing classifiers. Wang et al. [5] proposed a bag-of-words model for biomedical EEG and ECG time series that are represented as a histogram of the code words. The results of the proposed model are insensitive to the used parameters and are also robust to noise.

Feature extraction techniques proposed in the literature can generally be categorized into time-domain- or frequency-domain-based according to the features used. These techniques were used in several research works [10, 11].

Time-domain features are easily computed, and their time complexity is usually manageable [10]. Vidaurre et al. [12] proposed a time-domain-parameter- (TDP-) based feature extraction method. It is a generalized form of the Hjorth parameter and can be computed efficiently. The TDP feature is then fed to a linear discriminant analysis (LDA) classifier that is utilized in a brain computer interface application. Mohamed et al. [13] proposed five time-domain features, namely, sum, average, standard deviation, zero crossing, and energy. Subsequently, they used a set of classifiers to detect epileptic seizures. The output of the classifiers was then combined, using the Dempster rule of combination, for a final system decision. A classification accuracy of 89.5% was achieved. Nigam and Graupe [14] proposed an automated neural network-based epileptic seizure detection model, called LAMSTAR. Two features, namely, the relative spike amplitude and the spike rhythmicity of the EEG signals, were calculated and utilized to train the neural network.

Frequency-domain features are usually obtained by transforming EEG signals into their basic frequency components [6]. The characteristics of these components primarily fall within four frequency bands [15]. One classification system uses a one-second time window to extract relevant features [16]. The fast Fourier transformation (FFT) is used to transform the data in the window into the frequency domain. To distinguish between several brain states, frequency components from 9 to 28 Hz were studied and presented to a modified version of Kohonen's learning vector quantization classifier. Wang et al. [17] proposed an EEG classification system for epileptic seizure detection. It consists of three main stages, namely, (1) the best basis-based wavelet packet entropy method, which is used to represent EEG signals by wavelet packet coefficients; (2) a *k*-NN classifier with the cross-validation method in the training stage of hierarchical knowledge base (HKB) construction; and (3) the top-ranked discriminating rules from the HKB used in the testing stage to compute the classification accuracy and rejection rate. They reported a classification accuracy of close to 100%; however, their experiments considered only healthy subjects which is class A and epileptic seizure active subjects which is class E data and never considered seizure-free intervals which are class C or class D. Trivially, neglecting such classes eliminated the main source of difficulty in this classification process. Moreover, the data of their experiments is only noiseless and used a single classifier, *k*-NN. Bajaj and Pachori [18] proposed a new method for classifying seizure and nonseizure states. The method used the empirical mode decomposition (EMD) technique based on bandwidth features. The features were used as an input to a least squares SVM classifier. Sharma et al. [19] also presented a classification method of two focal and nonfocal EEG signals. Data from five epilepsy patients who had longstanding drug resistance has been used to test the method. The only base classifier used was the least square support vector machine (LS-SVM). Average sample entropy and average variance of the intrinsic mode functions (IMFs) were obtained based on EMD of EEG signals. The results show that the proposed method gives a classification accuracy of 85%. The second-order difference plot method of IMF [20] has been used as a feature for epileptic seizure classification. The computed area from the diagnostic signal demonstrates that the IMF detection is found to be a significant parameter for analysis of both healthy and unhealthy subjects [21]. The mean frequency feature of the IMFs has come up as a feature to identify variance between ictal and seizure-free EEG signals [22]. Wavelet and multiwavelet transformations have been included in analysis and classification of EEG time-frequency of the epileptic seizure [23]. However, these methods used noiseless data, while in this research both noiseless and noisy data were used. Compared with our methods, these datasets are only using the LS-SVM as a base classifier, while in this research 4 different classifiers were used. In another research work [24], the discrete wavelet transform (DWT) was used to transform EEG signals into their frequency components. For each wavelet subband, the maximum, minimum, mean, and standard deviation were then calculated and used as an input vector for a set of classifiers. The results revealed that the neural network classifier outperformed other classifiers with a 95% accuracy rate, while the *k*-NN classifier was more tolerant to imperfect data.

Other reported techniques utilize a mix of time- and frequency-domain features, such as in Valderrama et al. [25]. The first, second, third, and fourth statistical moments (i.e., mean, variance, skewness, and kurtosis, resp.) were extracted using the EEG amplitudes. Along with these time-domain features, energy and other frequency-domain features were extracted. A support vector machine (SVM) was then applied to the obtained features for seizure classification. Weng and Khorasani [26] proposed a method that uses the average EEG amplitude, average EEG duration, coefficient of variation, dominant frequency, and average power spectrum as features that are input to an adaptive structured neural network.

The classification techniques that are reported throughout the literature provide satisfactory performance data indicating that the EEG data are not contaminated by different factors. Although the raw EEG datasets (free of artifacts) were used, the lossy compression will introduce signal distortion, which will affect the reconstructed data. Therefore, wireless EEG data often are compressed before transmission, which means that some important information may get lost during the reconstruction process on the receiver side. Moreover, a wireless channel may augment the transmission problem by adding noise artifacts to the transmitted data. Therefore, a prospective classification technique should consider the uncertainty in the EEG data to guarantee the targeted performance.

In this paper, considering that the EEG signal is in nature bandwidth hungry, several works have considered in-network processing for either compressing EEG data [27] or transferring EEG features instead of delivering the raw uncompressed signal [28]. Another reason considering that the sensor is battery-operated, if the data is transmitted without compression, the battery power will be consumed faster. Therefore, we propose unified framework where the EEG data is compressed using compressive sensing (CS) and sent using two different types of channels. In the first, it was sent over a noiseless channel while the second was sent over the additive white Gaussian noise (AWGN) wireless channel in three different cases where SNR = 1, 5, and 10 dB. On the other hand, the compressed data was reconstructed and statistical features were extracted. Finally, the data obtained was contaminated due to the reconstruction and the different values of noise. A distinct factor that distinguishes this research work is the proposal of a new framework and new noise-aware signal combination (NSC) method that improves the classification of the reconstructed and noisy EEG data. To address this scenario, a unified framework has been designed, which presents compressive sensing-based technique to send compressed EEG data over AWGN wireless channel, reconstruction, and feature extraction using time-frequency-domain analysis in preparation of data classification. Such framework makes this work more practical because it performs classification considering data imperfection due to compression and wireless channel transmission.

Thus, the main contributions of this paper are as follows: (1) a framework for EEG compression and classification using CS and AWGN channel transmission has been developed, (2) a new noise-aware signal combination (NSC) method that supports both types of biomedical reconstructed EEG data, noiseless and noisy, has been proposed, and (3) a series of comprehensive experiments are conducted to investigate the effectiveness and robustness of the NSC method for classifying EEG signals.

The remainder of this paper is structured as follows. In Section 2, we present an EEG-based framework, including compressive sensing, the DCT method, and feature extraction, as well as the set of classifiers that have been used.  Section 3 describes the proposed system model, which mainly includes an ensemble classification method, a description of the EEG datasets, an epileptic seizure detection system model, and a proposed noise-aware signal combination (NSC) method. The results and discussions of extensive experiments investigating the effectiveness and robustness of NSC for EEG signal classification are illustrated in Section 4, and the paper is concluded in Section 5.

## 2. Materials and Methods

Firstly, this section describes the framework of the implemented system and its architecture as well as the main components. Secondly, a description of the EEG datasets, which is being used to distinguish between healthy subjects and epilepsy subjects, is presented. Thirdly, the compressive sensing integrated with the discrete cosine transform and measurement matrix is being presented. Fourthly, feature extraction in described, and finally, a brief of classification methods are demonstrated.

### 2.1. Architecture of the Framework

**Figure 1:**
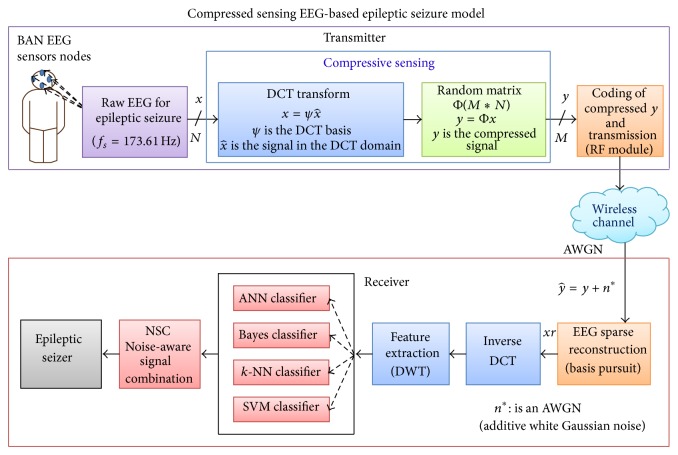
EEG-based epileptic seizure framework.

The system model is composed of two main parts, the transmitter and the receiver, shown in Figure 1. The transmitter has 4096 samples raw electroencephalography (EEG) signals, represented by (*x*), and uses a CS technique to downsample the data based on a sparse measurement matrix. In this framework, we used DCT and the basis *ψ* for different quantities of *M* to obtain the compressed data $\hat{x}$ that will be transmitted over noiseless and noisy channels (i.e., radio frequency (RF) or Bluetooth). Several sources of noise can alter the data, including wireless channel fading, path loss, and thermal noise at the receiver. In this paper, without loss of generality, we consider the thermal noise using the AWGN model at the receiving side as the most widely used model for representing thermal noise [29–32]. We control the noise level using the signal to noise ratio (SNR) to demonstrate data imperfection and to study the behavior of the different classification techniques in the presence of such noise.

The receiver, which receives the compressed signal of size *M*, reconstructs the EEG data using an inverse DCT (iDCT) and basis pursuit to obtain the reconstructed signal. The iDCT reconstruction algorithm is used for the DCT, or an optimization problem with certain constraints is solved for the CS [30, 33, 34]. For example, in the following, for a given compressed measurement *y* at the receiver, the signal *x* can be reconstructed by solving one of the following optimization problems:

(1) Minimum x02Subject  to  yi=ΦiΨx0i.

Using a trick of basis pursuit, find the vector *x*_0_ with the lowest *L*_2_ norm that satisfies the observations. For an *N*-dimensional EEG signal *x*,

(2) $$\begin{array}{c} x=\;\Psi \alpha , \end{array}$$

where Ψ is a discrete cosine transform (DCT) basis, *α* is the wavelet, and both are domain coefficients. At the receiver side, once we detect *α*, iDCT will be utilized to reconstruct the original signal from *α*. Figure 1 shows the framework that has compressive sensing and data reconstruction as well as the classification processes for EEG-based epileptic seizure [24].

### 2.2. EEG Datasets Descriptions

**Figure 2:**
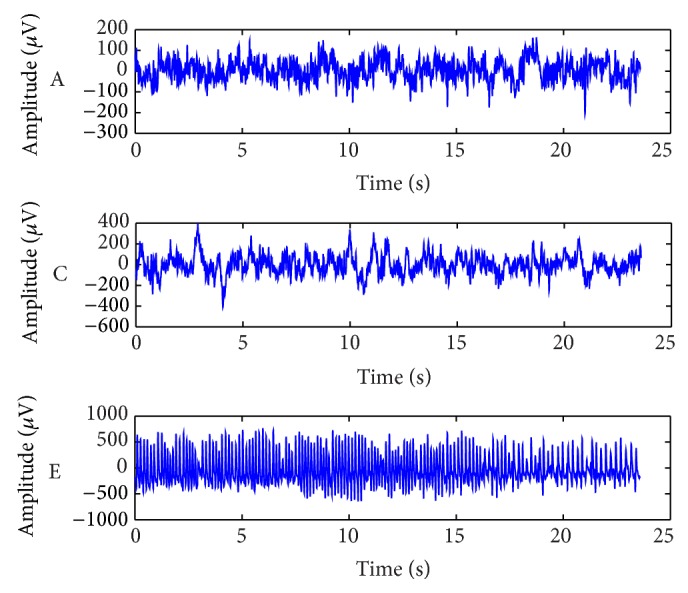
Example of three different classes of EEG signals taken from different subjects.

The datasets used in this work originated from Andrzejak et al. [35], which are widely used for automatic epileptic seizure detection. It contains both normal and epileptic EEG datasets. The EEG datasets were collected from five patients. The datasets consist of five sets termed A, B, C, D, and E. Each set was composed of 100 single channel EEG segments of 23.6-second duration. For sets A and B, the patients were relaxed and awake with eyes open and eyes closed, respectively. Segments of sets A and B were taken from surface EEG recordings, which were performed using a standardized electrode placement scheme on five healthy subjects. The segments in set C were recorded from the hippocampal formation of the opposite from the epileptogenic zone. The segments in set D were recorded from within the epileptogenic zone. Sets C, D, and E originated from EEG archive of presurgical diagnosis. Sets C and D both contained only the activity measured during seizure-free intervals. Finally, only set E contained seizure activity. All EEG signals were recorded with the same 128-channel amplifier system (neglecting electrodes that have strong eye movement artifacts (A and B) or pathological activity (C, D, and E)). The data were constantly written at a sampling rate of 173.61 Hz to the disk of the data acquisition computer system. Kumar et al. [36] reported that when the performance of sets A and E was compared with set B and set E, it was concluded that set A and set E were more efficient [36]. In addition, set A and set B are similar in feature properties that are hard for the classifier to distinguish between both sets representing healthy patients. It is worth noting that, during performance evaluation, we have conducted many experiments using different groups of classes (i.e., one group was all 5 classes; another group was A, C, and E, etc.), and the best results were evident for the class groups of A, C, and E. Therefore, in this research paper, we opted to use set A to represent healthy subjects, set C to represent unhealthy with seizure-free interval subjects, and set E to represent the epileptic seizure active subjects. In this case, 300 EEG segments are used; each class consists of 100 segments.  Figure 2 illustrates the ideal raw EEG signals of sets A, C, and E, respectively.

Typically, transmitters are mobile devices, which are equipped with battery sources; hence, the power consumption during data transmission is critical. Therefore, the compressive sensing (CS) and discrete cosine transform (DCT) methods have been utilized to reduce the amount of data before transmission because CS does not require much complexity for downsampling at the transmitter; this low complexity comes with the cost of higher complexity on the receiver side [29].

### 2.3. Compressive Sensing

Compressive sensing (CS) technique [37] is used to reduce the size of the data that was sent from the transmitter to the receiver, and thus CS has been considered for efficient EEG acquisition and compression in several applications [31, 38, 39]. Signal acquisition is the critical part of most applications, where the acquisition time or the computational resources are limited, and the CS technique has the significant advantage of offloading the processing from the data acquisition step to the data reconstruction step. CS reduces the time consumed in processing at the transmitter, at the expense of higher complexity at the receiver where more processing time and higher computational capacity are usually available. Previous research work [38, 39] focused on the sparse modeling of EEG signals and evaluating the efficiency of CS-based compression in terms of signal reconstruction errors and time required.

An *N*-dimensional 4096-sample raw EEG signal *x* is considered to illustrate the CS compression and reconstruction. Assume that this signal is represented by a projection onto a different basis set Ψ:

(3) $$\begin{array}{c} x=\overset{N}{\underset{i=1}{\sum }}{{x_{0}}_{i}\Psi }_{i}\;\text{or}\;x=\;\Psi x_{0}, \end{array}$$

where *x* is the original signal, *x*_0_ is the sparse of representation of *x*, and Ψ is an *N*∗*N* bases matrix.

The sparse vector *x*_0*i*_ can be calculated from the inner product of *x* and Ψ:

(4) $$\begin{array}{c} x_{0i}=\langle x,\Psi_{i}\rangle . \end{array}$$

The basis (Ψ) can be a Gabor, Fourier, or discrete cosine transform (DCT) or a Mexican hat, linear spline, cubic spline, linear B-spline, or cubic B-spline function. In compressive sensing, Ψ is chosen such that *x*_0_ is sparse. The vector *x*_0_ is *k*-sparse if it has *k* nonzero entries and the remaining (*N* − *k*) entries are all zeroes. In addition to the projection above, it is assumed that *x* can be related to another signal *y*:

(5) $$\begin{array}{c} y_{\left[M*1\right]}=\Phi_{\left[M*N\right]}\times x_{\left[N*1\right]}, \end{array}$$

where Φ is a measurement matrix (also called sensing matrix) of dimensions *M*∗*N*, and *y* is the compressive sensed version of *x*. Matrix *y* has dimensions *M*∗1, and data compression is achieved if *M* < *N*. It can be shown that this technique is possible if Φ and Ψ are incoherent. To satisfy this condition, Φ is chosen as a random matrix. The compression ratio (CR) is then defined as follows:

(6) $$\begin{array}{c} \text{CR}=\left(1-\frac{M}{N}\right)*100. \end{array}$$

### 2.4. Discrete Cosine Transom (DCT) Method

The discrete cosine transform (DCT) is used as the basis to make the EEG signal sparse as part of the CS framework. It is a Fourier-related transform similar to the discrete Fourier transform (DFT); however, it only uses real numbers and has low computational complexity [24, 28]. Obtaining the signal *x*(*n*) in the DCT domain will require a definition of the (*N* + 1)∗(*N* + 1) DCT transform matrix, whose elements are given by

(7) Cmn=2Nkmkncos⁡mnπN,      m,n=0,1,…,N,ki=1,for  i≠0  or  N,12,for  i=0  or  N. 

This matrix is unitary, and when it is applied to a data vector *x* of length *N* + 1, it produces a vector called *X*_*c*_, where *X*_*c*_ = [*C*]∗*x*, and its elements are given by

(8) $$\begin{array}{c} X_{c}\left(m\right)=\sqrt{\frac{2}{N}}\;\overset{N}{\underset{n=0}{\sum }}k_{m}k_{n}\cos\left(\frac{mn\pi }{N}\right)x\left(n\right). \end{array}$$

On the receiver side, the basis of the iDCT [28] is utilized in the CS decoder in order to obtain the reconstructed signal (*x*_*r*_) as follows:

(9) $$\begin{array}{c} x_{r}\left(a\right)=\overset{N}{\underset{k=1}{\sum }}w\left(k\right)y\left(k\right)\cos\left[\frac{\pi \left(2a+1\right)k}{2N}\right], \end{array}$$

where *N* is the length of both time series and cosine transform signals, *a* is the time series index (*a* = 1, 2,…, *N*), *k* is the cosine transform index (*k* = 1, 2,…, *N*), and the window function *W*(*k*) is defined as

(10) $$\begin{array}{c} w\left(k\right)=\left\{\begin{array}{cc} \frac{1}{\sqrt{N}}, & k=1,\\ \sqrt{\frac{2}{N}}, & 2\leq k\leq N. \end{array}\right. \end{array}$$

After obtaining the contaminated reconstructed signal (*x*_*r*_), DWT is used as feature extraction and selection techniques.

### 2.5. Feature Extraction

EEG feature extraction plays a significant role in diagnosing most brain diseases. Obtaining useful and discriminant features depends largely on the feature extraction method used. Because EEG signals are time-varying and space-varying nonstationary signals, the discrete wavelet transform (DWT) method is widely used [17]. It captures both frequency and time location information [32, 40–42]. Using multiresolution wavelet analysis, DWT basically decomposes the EEG signals into different frequency bands.

EEG data are generally nonstationary signals, which are heavily dependent on the subject condition. The Daubechies 6 DWT was employed, where the data were sampled at a rate of 173.61 Hz. This means that the EEG data frequency is 86.81 Hz; thus, the filter length is long as well; the frequency wavelet subband is the same as the fundamental component of the EEG. Hence, decomposition level 7 was calculated based on the EEG frequency. In addition, considering our extensive experimental work on the reconstruction accuracy of different wavelet families, filter lengths, and decomposition levels [30], we used Daubechies 6 with 1–8 different decomposition levels in this research. We found that Daubechies 6 with decomposition level 7 is the optimum level in terms of classification accuracy and computational complexity of the EEG epileptic seizure category of data. Given the EEG signal *f*(*x*), the wavelet series expansion is depicted [30] as follows:

(11) fx=∑kcj0kφj0,k(x) +∑j=jo∞∑kdjkψj,kx,

where *f*(*x*) ∈ *L* ^2^(*R*), *L* ^2^(*R*) is relative to the wavelet *ψ*(*x*) and the scaling function *φ*(*x*), and *c*_*j*0_ are the approximation coefficients.

In the first sum, the approximation coefficients *c*_*j*0_ can be represented as the outcome of the inner product process between the original signal *f*(*x*) and the approximation function *φ*_*j*0,*k*_(*x*) as expressed by

(12) $$\begin{array}{c} c_{j_{0}}\left(k\right)=\langle f\left(x\right),\varphi_{jo,k}\left(x\right)\rangle . \end{array}$$

In the second sum, a finer resolution is added to the approximation to provide increasing details. The function *d*_*j*_(*k*) represents the details coefficients and it can be obtained by the inner product between the original signal *f*(*x*) and the wavelet function *ψ*_*j*,*k*_(*x*) calculated as

(13) $$\begin{array}{c} d_{j}\left(k\right)=\langle f\left(x\right),\psi_{j,k}\left(x\right)\rangle . \end{array}$$

Generally, the classification accuracies improve when using a combination of time- and frequency-domain features rather than features solely based on either the frequency domain or the time domain [30]. Different implementation choices, including different wavelet families, filter lengths, and decomposition levels, have been utilized to extract features. Accordingly, the conventional statistical features (*maximum, minimum, mean, and standard deviation*) are extracted from each wavelet subband. The extraction rules for statistical features that have been implemented for the wavelet subband are as follows.

Maximum feature:

(14) $$\begin{array}{c} x_{k}\;\;\text{such}\;\text{that}\;\;x_{k}>x_{i},\;\forall i\neq k,\;\;i=1,\ldots ,n,\\ d_{i}\left(x\right)=\underset{i=1,\ldots ,k}{\max}\left\{d_{i}\left(x\right)\right\}. \end{array}$$

Minimum feature:

(15) $$\begin{array}{c} x_{k}\;\;\text{such}\;\text{that}\;\;x_{k}<x_{i},\;\forall i\neq k,\;\;i=1,\ldots ,n,\\ d_{i}\left(x\right)=\underset{i=1,\ldots ,k}{\min}\left\{d_{i}\left(x\right)\right\}. \end{array}$$

The mean can be calculated by the following:

(16) $$\begin{array}{c} \hat{x}=\frac{1}{N}\overset{n}{\underset{i=1}{\sum }}x_{i}. \end{array}$$

The standard deviation feature is given by the following:

(17) $$\begin{array}{c} \sigma^{2}=\frac{1}{N-1}\overset{N}{\underset{i=1}{\sum }}{\left(x_{i}-\hat{x}\right)}^{2}. \end{array}$$

The original EEG signal was analyzed for the wavelet subbands A7 and D7-D1. Eventually, four conventional statistical features are selected from each wavelet subband individually. As a consequence, 32 attributes are obtained from the whole subbands to be fed to the classifiers. So the features* maximum, minimum, mean, and standard deviation* contribute to the classification accuracy in this research. It has been found that these features are robust with the dynamic environment of the wireless channel [24, 28]. Meanwhile, these features have low computational complexity.

### 2.6. Classification Methods

EEG detection and classification play an essential role in timely diagnoses and analyze potentially fatal and chronic diseases proactively in clinical as well as various life settings [3, 43]. Liang et al. [44] proposed a systematic evaluation of EEGs by combining both complexity analysis and spectral analysis for epilepsy diagnosis and seizure detection. Approximately 60% of the features extracted from the dataset were used for training, while the remaining ones were used to test the performance of the classification procedure on randomly selected EEG signals [44].

In this research work, four different classifiers have been used, namely, ANN, naïve Bayes,* k*-NN, and SVM. Initially, the classifiers were developed to work individually to compare their performances. However, we developed a data fusion method for combining the output of all classifiers in order to reduce the effect of data imperfections while maximizing the classification accuracy. Each classifier belongs to a different family of classifiers and has been shown to be the best classifier in its family. However, it is expected that they may yield different classification results because they each use a different classification strategy [13, 17, 45–47]. The following provides a brief description of these classifiers.

#### 2.6.1. Artificial Neural Network

An artificial neural network (ANN) is a mathematical model that is motivated by the structure and functional aspects of biological neural networks. To establish classification rules and perform statistical analysis, ANN is able to estimate the posterior probabilities [14, 47, 48]. The ANN has several parameters; in this paper, the ANN configuration uses training cycles = 500, learning rate = 0.3, and momentum decay = 0.2.

#### 2.6.2. Naïve Bayes

The naïve Bayes (NB) classifier is a statistical classifier. It is a simple probabilistic classifier based on the application of Bayes'theorem. The NB method involves an assumption that makes the calculation of the NB classifiers more efficient than the exponential complexity. Simply, it works by considering that the presence of certain features of a class is irrelevant to any other features. The NB classifier considers each feature independently to calculate the feature properties that contribute to the probability of a certain class to be the outcome of the classification. It then uses Laplace correction to prevent high encounters of zero probabilities as the default configuration [13, 24, 46].

#### 2.6.3. k-Nearest Neighbor (k-NN)

The *k*-nearest neighbor (*k*-NN) algorithm compares a given test sample with training samples that are alike, where *k* parameter is a small positive and odd integer value. This algorithm combines two steps. First, find the *k* training samples that are closest to the invisible sample. Second, take the commonly occurring classification for these *k* samples and find the average of the values of its *k-*nearest neighbors in the regression. It can be defined by a distance metric called the normalized Euclidean distance, as indicated in the following equation, given two points *Y*_1_ = (*y*_11_, *y*_12_,…, *y*_1*n*_) and *Y*_2_ = (*y*_21_, *y*_22_,…, *y*_2*n*_) [6, 24, 45]:

(18) $$\begin{array}{c} \mathrm{dist}\left(Y_{1},Y_{2}\right)=\sqrt{\overset{n}{\underset{i=1}{\sum }}{\left(y_{1i}-y_{2i}\right)}^{2}}. \end{array}$$

In this research, the *k*-NN configuration uses value of *k* = 10, and* mixed measures* were selected as the measure type, which makes the* mixed Euclidean distance* the only available option.

#### 2.6.4. Support Vector Machine

The support vector machine (SVM) learner is a strong classifier based on statistical learning theory. SVM constructs an ideal hyperplane in order to separate the data into two different classes to minimize the risks. SVM takes a set of input data and predicts, for each given input, which of the two possible classes involves the input. SVM is an integrated and powerful method for both classification and regression as well as distribution estimation. SVM operator supports types* C-SVC* and* nu-SVC* for classification tasks;* epsilon-SVR* and* nu-SVR* types for regression tasks. Finally, the* one-class* type is used for distribution estimation [13, 24, 46, 49]. In this research, SVM configuration is consist of both* nu-SVC* and* radial basis function* kernel were used for SVM configurations consist of both classification technique.

## 3. Ensemble Detection and Classification

Ensemble methods are introduced first, followed by the proposed ensemble system model and, finally, the ensemble method in this section.

### 3.1. Ensemble Classification Methods

Several combination techniques have been introduced in the literature, and each offers certain advantages and suffers from certain limitations. However, given several classifiers, the combination (fusion) method must address two critical issues: the dependency among the potentially combined classifiers and the consistency of the information contained in each classifier.

For the first issue, the classifiers must be independent because we consider each classifier to be a source of information. This means that each classifier simply works on the input feature set independently, while the classification is based on combining the outcomes of all classifiers simultaneously.

For the second issue, the classifiers may have conflicting decisions because different classifiers are expected to consider different viewpoints of the current system state. To address this anticipated conflict, an effective mechanism that is capable of quantifying the assurance in the decision of each classifier is desirable.

One of these well-known combination techniques is the majority voting. The majority voting (MV) rule technique collects the votes of all classifiers and investigates the class name that is mostly reported by the classifiers. It then chooses that class as a final decision [50]. However, MV is based on the idea that the classifiers participating in the voting process have the same weight. It completely ignores the inconsistency that may arise among the classifiers. This, of course, can cause less capable classifiers to override more capable classifiers. Thus, the performance of the classification system can be deteriorated. Because the classifier models proposed in this work are expected to have different discriminant weights, the MV technique is not suitable as a combination method.

In contrast, in probability-based voting schemes, the combination method should assign a probability value (*p*) that reflects the confidence of a classifier in its viewpoint. One of these schemes can be based on an accumulated experience. For instance, a given classifier is correct in identifying a certain hypothesis 75% percent of the time, while another classifier can correctly identify a different hypothesis 30% of the time. These values can actually be interpreted as probability assignments.

If the classifiers happen to provide these different and conflicting hypotheses as an explanation of the current system state, then the classifiers should not be treated equally at the classification stage. Clearly, the first classifier is more confident in its decision than the first one. This valuable information should be incorporated into the fusion (combination) process.

For instance, we may assign a weight (*p*) of 0.75 to the first classifier while assigning only 0.30 as a weight to the second classifier.

Let *T* be the set of classifiers:

(19) $$\begin{array}{c} T=\left\{C_{1},C_{2},\ldots ,C_{n}\right\}, \end{array}$$

and let *C* be the set of classes:

(20) $$\begin{array}{c} \left\{p_{1},p_{2},\ldots ,p_{n}\right\}. \end{array}$$

Then, let *d*_*i*,*j*_ be the decision of the classifier *i* and have the following definition:

(21) $$\begin{array}{c} d_{i,j}\in \left\{0,1\right\}, \end{array}$$

where  *i* = 1,…, *T* and *j* = 1,…, *C*.

Let *p*_*i*_ represent the weight of the classifier *i*. Then, the probability-based voting decision is calculated as

(22) $$\begin{array}{c} \overset{\left|T\right|}{\underset{i=1}{\sum }}p_{i}d_{i,j}=\begin{array}{c} C\\ \max\\ j=1 \end{array}\overset{\left|T\right|}{\underset{r=1}{\sum }}p_{r}d_{r,j}. \end{array}$$

Considering the weight of each classifier, (22) counts the votes from the participating classifiers.

### 3.2. Proposed Ensemble System Model

**Figure 3:**
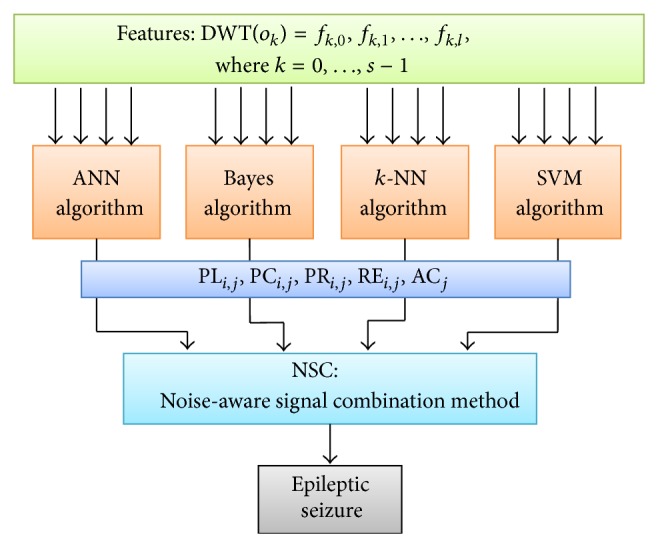
Proposed ensemble system.

The proposed model consists of three stages for detecting electroencephalogram seizures, namely, statistical feature extraction, classifier prediction, and proposed noise-aware signal combination (NSC) method. The extraction of statistical features was discussed in Section 2.5. For classifier prediction, four classifiers are utilized in this model, namely, ANN, Bayes, *k*-NN, and SVM. These classification methods are trained using the most popular data mining tools that are an industry standard and widely used tools for research. The training process is conducted on similar data adhering to various combinations of SNRs and downsampling rates. After exhaustive iterated experiments, the trained models are saved, and their averaged performances in different scenarios are reported to the NSC. The NSC is our proposed ensemble method using combinations of probability estimates. Eventually, the ultimate classification accuracy is obtained for the epileptic seizure detection. The proposed system model is shown in Figure 3.

There are *s* tabular observations *O* = {*o*_0_, *o*_1_,…,*o*_*s*−1_}, where each *o*_*i*_ is a *t*-tuple of readings *R*_*i*_ = (*r*_0_, *r*_1_,…,*r*_*t*−1_). These observations fall into (*s*/*m*) different categories of classes = {*c*_0_, *c*_1_,…, *c*_*m*−1_}.

The DWT is applied to the set of observations *O* to obtain an *l*-tuple of features *R*_*i*_ = (*f*_0_, *f*_1_,…, *f*_*l*−1_) for each *o*_*i*_ ∈ *O*. In other words, DWT : *O* → *F* such that DWT(*o*_*k*_) = (*f*_*k*,0_, *f*_*k*,1_,…, *f*_*k*,*l*_), where *f*_*k*,*j*_ is an *l*-tuple extracted feature for the observation *o*_*k*_ obtained by DWT.

Hence, DWT(*O*) = {(*f*_*i*,0_, *f*_*i*,1_,…, *f*_*i*,*l*_∣*i* = 0, 1, …, *s* − 1)} is the training and testing tabular *l*-tuple format representing the input data for the classification model in this research work.

### 3.3. The Ensemble Method

Several classifiers (*n*) built on various hypotheses *H* = {*h*_0_, *h*_1_,…, *h*_*n*−1_} are fed with input data. The data are DWT(*O*) in a tabular *l*-tuple format, as discussed above. Each classifier *k* built on hypothesis *h*_*k*_ is trained on the data to predict the label representing the class *c*_*j*_ that best describes a given set of features (*f*_*i*,0_, *f*_*i*,1_,…, *f*_*i*,*l*_) corresponding to the observation *o*_*i*_.

At the end of the training of each classifier, a set of performance measurements of interest is recorded.  Table 1 shows some of these performance measurements. The trained model will then be saved for application to various categories of testing data. This process is replicated and repeated to yield an output that can be averaged to describe the model behavior for long run times.

The proposed ensemble classification method is fed with the output of the *m* trained classifiers. In a sense, the training data are bundled first into two parts and are used to train the *m* classifiers on the patterns within each bundle. Finally, the classification decision of a testing sample is obtained from an ensemble of the decisions from the corresponding *m* classifiers at each layer using the noise-aware signal combination method. A subset of the performance measures of each classifier together with the predicted class label *c* ∈ *C* for an observation *o* ∈ *O* provided by each classifier with hypothesis *h* ∈ *H* are the input to the hypothesis used by this combined classifier.

The confusion matrix for each hypothesis *h*_*k*_ based on the reported performance results of the trained hypothesis *h*_*k*_ is calculated using the algorithm shown in Algorithm 1. An entry MRP (*i*, *j*) in the matrix of reported performance results for hypothesis *h*_*k*_ represents the frequency of predicting class *j*as being class *i*. Therefore, MRP (*i*, *i*) represents the frequency of correct predictions being in class *i*, while ∑_*j*≠*i*_^*m*^MRP (*i*, *j*) is the frequency of wrong predictions of other classes that are in class *i*.

Hence, PR_*i*_, the precision of class *i*, is (MRP (*i*, *i*)/∑_*j*≠*i*_^*m*^MRP  (*i*, *j*))%, and RE_*i*_, the recall of class *j*, is (MRP  (*i*, *i*)/∑_*i*≠*j*_^*m*^MRP  (*i*, *j*))%. Finally, AC_*i*_, the accuracy using hypothesis *h*_*k*_, is the averaged precision of the classes and is given by (∑_*i*_^*m*^ *PR*_*i*_/*m*) × 100%.

In the confusion matrix, an entry CM (*i*, *j*) is the weighted entry MRP (*i*, *j*) on class *i* recall. That is, CM  (*i*, *j*) = MRP  (*i*, *j*)/∑_*i*_^*m*^MRP  (*i*, *j*), where ∑_*i*_^*m*^CM (*i*, *j*) = 1. The ${\overset{-}{\text{PR}}}_{i}$ in the confusion matrix is the weighted PR_*i*_ across the set of hypotheses *H*given by PR_*i*_/∑_*j*_^*n*^PR_*i*_, where $\sum_{i}^{n}{\overset{-}{\text{PR}}}_{i}\;=1$. ${\overset{-}{\text{RE}}}_{i}$ and ${\overset{-}{\mathrm{AC}}}_{i}$ are also weighted across *H* and are calculated in the same manner.

The prediction of the combined classifier is calculated following the hypothesis with the highest probability calculated as

(23) $$\begin{array}{c} P\left(h_{j}\right)=\frac{\sigma \left({\overset{-}{\text{PR}}}_{k,j}+{\overset{-}{\text{RE}}}_{k,j}\right)+\left(1-\sigma \right){\overset{-}{\mathrm{AC}}}_{j}}{\sum_{i=0}^{n-1}\sigma \left({\overset{-}{\text{PR}}}_{k,i}+{\overset{-}{\text{RE}}}_{k,i}\right)+\left(1-\sigma \right){\overset{-}{\mathrm{AC}}}_{i}}, \end{array}$$

where *k* is the label of the predicted class and ∑_*j*=0_^*n*−1^ *P*(*h*_*j*_) = 1.

Tables 2 [Table tab3] [Table tab4]–5 show the confusion matrices for the four classifiers, namely, ANN, naïve Bayes, *k*-NN, and SVM. These matrices represent the finalized weighted performance of the trained classifiers based on noiseless data and three different levels of data noise, SNR = 1 dB, 5 dB, and 10 dB, for EEG-based epileptic seizures at *M* = 600 downsampling. Also, these tables show that *c*_0_, *c*_1_, and *c*_2_ are representing class A, class C, and class E, respectively.

For example, Table 2 represents noiseless EEG data, classes *c*_0_, *c*_1_, and *c*_2_ in vertical line are representing the predicted class label; on the other hand, in the horizontal line, we show the true class label. The normalized precision $\overset{-}{\text{PR}}$ of class A in the first row of the four matrices is 0.273, 0.252, 0.226, and 0.249 for ANN, NB, *k*-NN, and SVM, respectively. The normalized class recall $\overset{-}{\text{RE}}$ of class A in the first four matrices is 0.247, 0.253, 0.270, and 0.230 for ANN, NB, *k*-NN, and SVM, respectively. Furthermore, the normalized overall accuracy $\overset{-}{\mathrm{AC}}$ is 0.259, 0.254, 0.239, and 0.248 for the same set of classifiers, respectively.

At the end of each experiment, the algorithm calculates the performance of each classifier, based on the recorded test results. The next section reports the obtained results and provides illustrations and discussions relevant to the performance of NSC compared with that of the other individual classifiers.

## 4. Results and Discussion

This research work addresses EEG-based epileptic seizure data classification considering noiseless and noisy data with different values of SNR. For each point on the graphs, we have conducted 10 experiments and calculated the average accuracy and its standard deviation accordingly. The standard deviation describes the distribution range, describing how much difference occurs between successful computations, which correspond to the data imperfection. In this case, the standard deviation (SD) is important to show the difference between successive measurements to make sure that the classifiers are not affected by data imperfection.  Table 6 shows the calculated performance measures of the studied classifiers with EEG-epileptic seizure data compressed with CR = 85.35% for noiseless and added noise of SNR = 1, 5, and 10 dB. The class precision (PR), class recall (RE) and the classification average (AVG) accuracy (AC), and standard deviation (STD) for each classifier for different SNR and noiseless channel conditions are also shown in Table 6.

**Figure 4:**
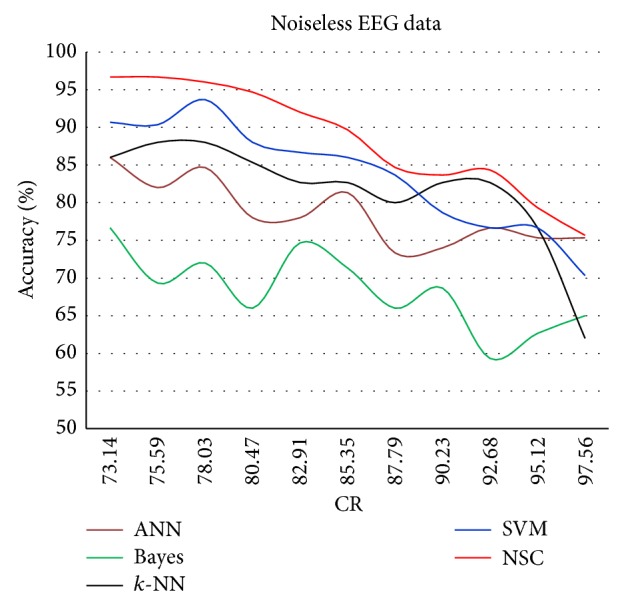
Classification accuracy against CR for noiseless data.

**Figure 5:**
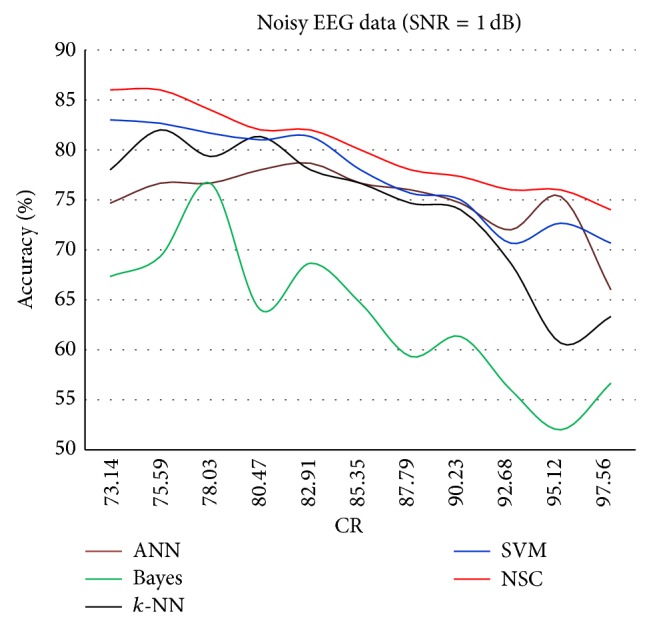
Classification accuracy against CR for SNR = 1 dB.

**Figure 6:**
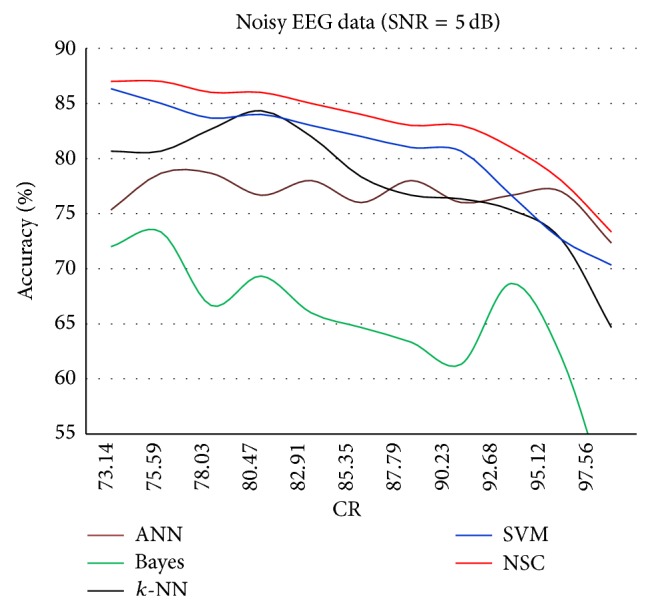
Classification accuracy against CR for SNR = 5 dB.

**Figure 7:**
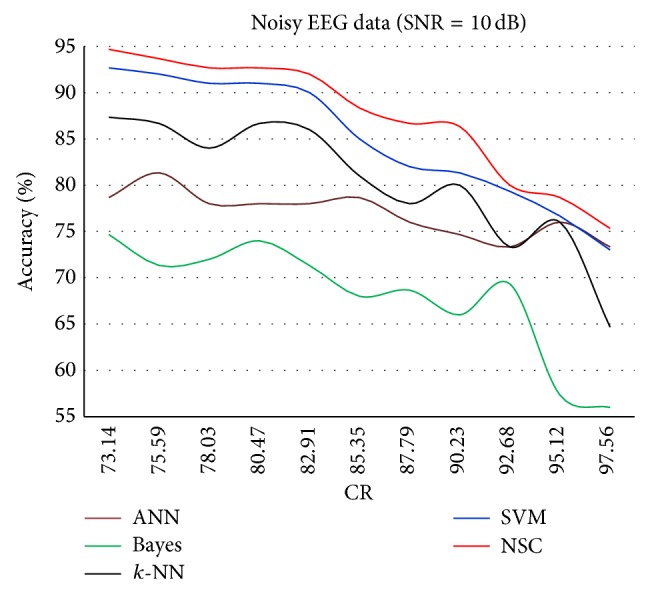
Classification accuracy against CR for SNR = 10 dB.

**Figure 8:**
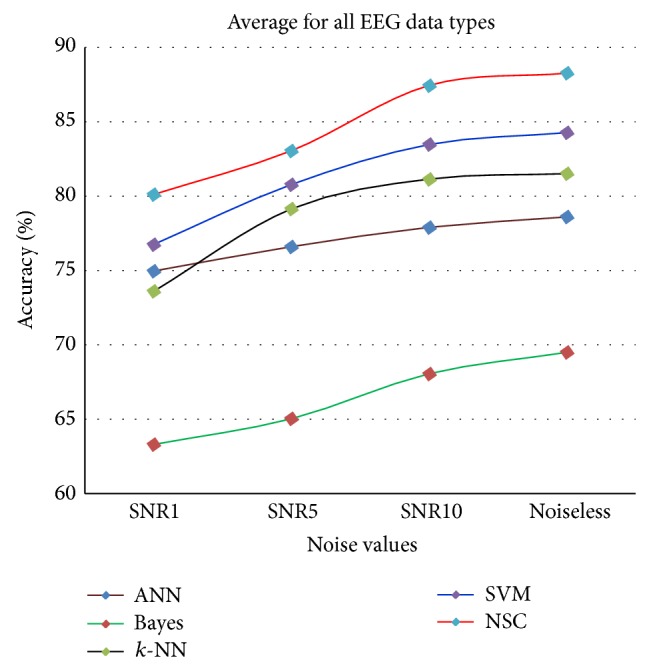
Average accuracy of classifiers for all CR values with SNR = 1, 5, and 10 dB and noiseless EEG data.

The results for each of the individual classifiers ANN, NB, *k*-NN (with *k* = 10), and SVM in each SNR case together with the results of NSC are plotted to illustrate the differences in their performances. Figures 4–8 show the performance for noiseless and SNRs of 1, 5, and 10 dB, respectively. The corresponding accuracies in Table 6 are emphasized in Figures 4–7 with the line drawn at CR = 85.35%. The constraint on the desired accuracy in the case of noiseless data is to achieve 90%. The CR of 84.35% was the cutting edge of achieving this desired goal. Therefore, the performance of the classifiers at this CR value is the most important to us. The overall accuracy results of all of the experiments show that this constraint is met at CR = 85.35%, while a high accuracy of 80% was maintained with very noisy data at SNR = 1 dB.

The results in Figure 4 show a trend in which the classification accuracy increases almost linearly with the decrease in CR. The decay in the accuracy seems to be reasonable in all regions, and NSC has the best accuracy, which starts to decay exponentially similar to the accuracy of all of the other individual classifiers.

Figure 5 shows the lower accuracies for all classifiers because of the injected quantity of AWGN (SNR = 1 dB), which is the highest noise injected in all experiments. In this case, the NSC continued to perform consistently better than the rest of the classifiers. In addition, the Bayes classifier continues to exhibit the poorest performance. The exact reported results at CR = 85.35% can be observed in Table 6.

As expected, Figure 6 shows that increasing the CR results in decreased overall accuracy for all classifiers.

Finally, Figure 7 shows a slightly different behavior for all classifiers. The classification accuracy of 90% starts to decay after CR = 82.91%. The effect of the AWGN is much less when SNR = 10 dB, which is close to the EEG data. To the best of our knowledge, no reported work has been found that employ similar evaluation approach of EEG-based epileptic seizure in which AWGN considers different SNR values. Moreover, new interesting results could be realized that the thermal noise using AWGN clearly affects the classification accuracy.

Overall and regardless of the compression ratio value, Figure 8 shows the results for the average classification accuracy; the NSC accuracy is constantly better than the accuracy of any individual classifier. This statement is valid for both noiseless and noisy EEG-epileptic seizure data.

Compared with previous works, the proposed NSC classification accuracy of noiseless EEG data has achieved 90%, which is 5% higher than the accuracy done in Sharma [19], 4.1% higher than the work done in Sadati et al. [15] (85.9% accuracy) especially for sets A, D, and E, and 0.5% higher than that reported in Mohamed et al. [13] (89.5% accuracy). In addition, Liang et al. achieved classification accuracy between 80% and 90% [44]. Tzallas et al. [11] achieved 89% only for noiseless dataset using one classifier. All of those approaches considered the same EEG dataset. In contrast to these methods, the proposed method achieved the desired and improved classification accuracy with noisy data using different SNR values: 80% for SNR = 1 dB, 84% for SNR = 5 dB, and 88% for SNR = 10 dB. These results were obtained at a CR of 85.35%. Moreover, the proposed method provides several significant benefits such as simplicity and the improvement of the overall classification accuracy.  Table 7 shows the comparisons between the proposed NSC and others reported in the literature.

## 5. Conclusion

In this paper, an EEG noise-aware signal combination method for EEG-based epileptic seizure detection applications is proposed and investigated. Compression paradigms with low complexity are achieved by utilizing the iDCT method for data reconstruction. Features are extracted from the reconstructed data using DWT. The proposed noise-aware signal combination (NSC) method together with the classifiers ANN, naïve Bayes, *k*-NN, and SVM is tested with different categories of EEG-based epileptic seizure data. Noise is introduced to the data at different levels: SNRs of 1, 5, and 10 dB. The proposed NSC combination method constantly performs better than any of the above four classifiers. The experimental results show that the proposed NSC technique is effective with noisy data of 80% for SNR = 1 dB, 84% for SNR = 5 dB, and 88% for SNR = 10 dB while being effective with 90% accuracy for noiseless data. These results were obtained at CR = 85.35%.

## Figures and Tables

**Algorithm 1:**
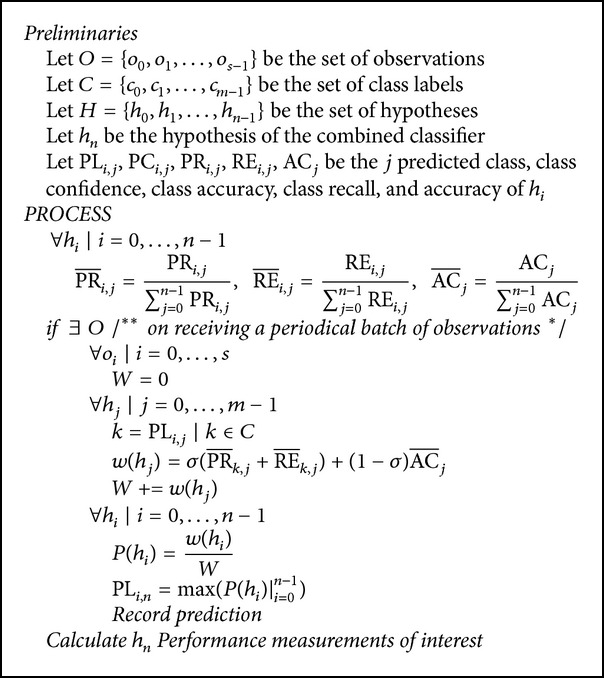
Noise-aware signal combination algorithm.

**Table 1:** Classifier's performance measurements.

Measure	Description
PL_*i*,*j*_	Predicted label of *o*_*i*_ using hypothesis *h*_*j*_
PC_*i*,*j*_	Confidence value predicting *c*_*i*_ using hypothesis *h*_*j*_
PR_*i*,*j*_	Precision value of *c*_*i*_ using hypothesis *h*_*j*_
RE_*i*,*j*_	Class recall value of *c*_*i*_ using hypothesis *h*_*j*_
AC⁡_*j*_	Accuracy value of for applying hypothesis *h*_*j*_

**(a):** 

	True class label			
	*c*_0_	*c*_1_	*c*_2_	$\overset{-}{\text{PR}}$
Predicted class label				
*c*_0_	0.88	0.12	0.02	0.273
*c*_1_	0.12	0.85	0.02	0.252
*c*_2_	0.00	0.03	0.96	0.245
$\overset{-}{\text{RE}}$	0.247	0.288	0.251	

*h*_0_ = ANN		$\overset{-}{{\mathrm{AC}}_{0}}$	0.259	

**(b):** 

	True class label			
	*c*_0_	*c*_1_	*c*_2_	$\overset{-}{\text{PR}}$
Predicted class label				
*c*_0_	0.90	0.23	0.00	0.252
*c*_1_	0.10	0.75	0.01	0.256
*c*_2_	0.00	0.02	0.99	0.248
$\overset{-}{\text{RE}}$	0.253	0.254	0.258	

*h*_1_ = NB		$\overset{-}{{\mathrm{AC}}_{0}}$	0.254	

**(c):** 

	True class label			
	*c*_0_	*c*_1_	*c*_2_	$\overset{-}{\text{PR}}$
Predicted class label				
*c*_0_	0.96	0.37	0.01	0.226
*c*_1_	0.04	0.63	0.09	0.244
*c*_2_	0.00	0.00	0.90	0.253
$\overset{-}{\text{RE}}$	0.270	0.214	0.235	

*h*_2_ = *k*-NN		$\overset{-}{{\mathrm{AC}}_{0}}$	0.239	

**(d):** 

	True class label			
	*c*_0_	*c*_1_	*c*_2_	$\overset{-}{\text{PR}}$
Predicted class label				
*c*_0_	0.97	0.19	0.07	0.249
*c*_1_	0.03	0.81	0.12	0.248
*c*_2_	0.00	0.00	0.81	0.253
$\overset{-}{\text{RE}}$	0.230	0.244	0.256	

*h*_3_ = SVM		$\overset{-}{{\mathrm{AC}}_{0}}$	0.248	

**(a):** 

	True class label			
	*c*_0_	*c*_1_	*c*_2_	$\overset{-}{\text{PR}}$
Predicted class label				
*c*_0_	0.88	0.38	0.04	0.249
*c*_1_	0.12	0.61	0.02	0.267
*c*_2_	0.00	0.01	0.94	0.249
$\overset{-}{\text{RE}}$	0.257	0.251	0.255	

*h*_0_ = ANN		$\overset{-}{{\mathrm{AC}}_{0}}$	0.257	

**(b):** 

	True class label			
	*c*_0_	*c*_1_	*c*_2_	$\overset{-}{\text{PR}}$
Predicted class label				
*c*_0_	0.82	0.40	0.00	0.248
*c*_1_	0.18	0.59	0.00	0.252
*c*_2_	0.00	0.01	1.00	0.249
$\overset{-}{\text{RE}}$	0.239	0.243	0.271	

*h*_1_ = NB		$\overset{-}{{\mathrm{AC}}_{0}}$	0.217	

**(c):** 

	True class label			
	*c*_0_	*c*_1_	*c*_2_	$\overset{-}{\text{PR}}$
				
Predicted class label				
*c*_0_	0.92	0.46	0.00	0.246
*c*_1_	0.08	0.53	0.07	0.256
*c*_2_	0.00	0.01	0.93	0.249
$\overset{-}{\text{RE}}$	0.268	0.218	0.252	

*h*_2_ = *k*-NN		$\overset{-}{{\mathrm{AC}}_{0}}$	0.264	

**(d):** 

	True class label			
	*c*_0_	*c*_1_	*c*_2_	$\overset{-}{\text{PR}}$
Predicted class label				
*c*_0_	0.81	0.30	0.05	0.257
*c*_1_	0.19	0.70	0.13	0.225
*c*_2_	0.00	0.00	0.82	0.252
$\overset{-}{\text{RE}}$	0.236	0.288	0.222	

*h*_3_ = SVM		$\overset{-}{{\mathrm{AC}}_{0}}$	0.262	

**(a):** 

	True class label			
	*c*_0_	*c*_1_	*c*_2_	$\overset{-}{\text{PR}}$
Predicted class label				
*c*_0_	0.94	0.31	0.02	0.260
*c*_1_	0.06	0.68	0.03	0.267
*c*_2_	0.00	0.01	0.95	0.249
$\overset{-}{\text{RE}}$	0.320	0.192	0.529	

*h*_0_ = ANN		$\overset{-}{{\mathrm{AC}}_{0}}$	0.258	

**(b):** 

	True class label			
	*c*_0_	*c*_1_	*c*_2_	$\overset{-}{\text{PR}}$
Predicted class label				
*c*_0_	0.94	0.36	0.00	0.254
*c*_1_	0.06	0.62	0.00	0.275
*c*_2_	0.00	0.02	1.00	0.247
$\overset{-}{\text{RE}}$	0.252	0.172	0.391	

*h*_1_ = NB		$\overset{-}{{\mathrm{AC}}_{0}}$	0.258	

**(c):** 

	True class label			
	*c*_0_	*c*_1_	*c*_2_	$\overset{-}{\text{PR}}$
				
Predicted class label				
*c*_0_	0.91	0.50	0.00	0.227
*c*_1_	0.09	0.50	0.011	0.216
*c*_2_	0.00	0.00	0.89	0.252
$\overset{-}{\text{RE}}$	0.313	0.252	0.471	

*h*_2_ = *k*-NN		$\overset{-}{{\mathrm{AC}}_{0}}$	0.231	

**(d):** 

	True class label			
	*c*_0_	*c*_1_	*c*_2_	$\overset{-}{\text{PR}}$
Predicted class label				
*c*_0_	0.89	0.22	0.10	0.259
*c*_1_	0.11	0.78	0.08	0.243
*c*_2_	0.00	0.00	0.82	0.252
$\overset{-}{\text{RE}}$	0.306	0.185	0.575	

*h*_3_ = SVM		$\overset{-}{{\mathrm{AC}}_{0}}$	0.252	

**(a):** 

	True class label			
	*c*_0_	*c*_1_	*c*_2_	$\overset{-}{\text{PR}}$
Predicted class label				
*c*_0_	1.00	0.52	0.00	0.263
*c*_1_	0.00	0.48	0.12	0.252
*c*_2_	0.00	0.00	0.88	0.248
$\overset{-}{\text{RE}}$	0.235	0.275	0.264	

*h*_0_ = ANN		$\overset{-}{{\mathrm{AC}}_{0}}$	0.257	

**(b):** 

	True class label			
	*c*_0_	*c*_1_	*c*_2_	$\overset{-}{\text{PR}}$
Predicted class label				
*c*_0_	0.86	0.38	0.08	0.252
*c*_1_	0.14	0.62	0.36	0.260
*c*_2_	0.00	0.00	0.56	0.246
$\overset{-}{\text{RE}}$	0.246	0.240	0.272	

*h*_1_ = NB		$\overset{-}{{\mathrm{AC}}_{0}}$	0.254	

**(c):** 

	True class label			
	*c*_0_	*c*_1_	*c*_2_	$\overset{-}{\text{PR}}$
Predicted class label				
*c*_0_	1.00	0.22	0.08	0.232
*c*_1_	0.00	0.78	0.12	0.234
*c*_2_	0.00	0.00	0.80	0.253
$\overset{-}{\text{RE}}$	0.262	0.206	0.239	

*h*_2_ = *k*-NN		$\overset{-}{{\mathrm{AC}}_{0}}$	0.237	

**(d):** 

	True class label			
	*c*_0_	*c*_1_	*c*_2_	$\overset{-}{\text{PR}}$
Predicted class label				
*c*_0_	1.00	0.30	0.02	0.253
*c*_1_	0.00	0.66	0.00	0.253
*c*_2_	0.00	0.04	0.98	0.253
$\overset{-}{\text{RE}}$	0.257	0.279	0.225	

*h*_3_ = SVM		$\overset{-}{{\mathrm{AC}}_{0}}$	0.252	

**Table 6:** Performance of the classifiers with CR = 85.35% for SNR = 1, 5, and 10 dB and noiseless EEG data.

	SNR	PR			RE			AC	
		*c*_0_	*c*_1_	*c*_2_	*c*_0_	*c*_1_	*c*_2_	AVG	STD
ANN	1	0.66	0.73	0.99	0.80	0.61	0.94	0.78	0.17
	5	0.74	0.88	0.99	0.84	0.68	0.95	0.82	0.14
	10	0.80	0.85	0.98	0.88	0.75	0.96	0.86	0.11
	Noiseless	0.86	0.82	0.92	0.84	0.82	0.96	0.87	0.08

NB	1	0.67	0.75	0.99	0.80	0.59	0.96	0.78	0.19
	5	0.71	0.79	0.98	0.88	0.62	0.96	0.82	0.18
	10	0.76	0.83	0.97	0.90	0.69	0.96	0.85	0.14
	Noiseless	0.80	0.87	0.98	0.90	0.75	0.99	0.86	0.12

*k*-NN	1	0.64	0.73	0.99	0.92	0.53	0.93	0.77	0.23
	5	0.65	0.71	1.00	0.91	0.50	0.89	0.78	0.21
	10	0.71	0.80	1.00	0.98	0.59	0.87	0.81	0.20
	Noiseless	0.72	0.83	1.00	0.96	0.63	0.90	0.83	0.18

SVM	1	0.70	0.69	1.00	0.81	0.70	0.82	0.78	0.07
	5	0.73	0.78	1.00	0.87	0.78	0.82	0.82	0.05
	10	0.76	0.84	1.00	0.94	0.80	0.82	0.85	0.08
	Noiseless	0.79	0.84	1.00	0.97	0.81	0.81	0.86	0.09

NSC	1	0.65	0.86	0.98	0.92	0.48	1.00	**0.80**	0.28
	5	0.70	0.93	0.98	0.96	0.56	1.00	**0.84**	0.24
	10	0.77	1.00	0.94	1.00	0.64	1.00	**0.88**	0.21
	Noiseless	0.83	0.95	0.94	0.98	0.74	0.98	**0.90**	0.14

**Table 7:** Comparisons between previous works and the proposed method.

Authors	Noiseless data	Imperfect data	Classifiers	Accuracy
Sharma et al., 2014 [19]	Two different classes	**N/A**	LS-SVM	85%
Sadati et al., 2006 [15]	A, D, and E	**N/A**	SVM, FBNN, ANFIS, and proposed ANFN	85.9%
Mohamed et al., 2013 [13]	A, B, C, D, and E	**N/A**	NB, MLP, *k*-NN, LDA, and SVM	89.5%
Liang et al., 2010 [44]	A, D, and E	**N/A**	PCA and GA	80%–90%
Tzallas et al., 2009 [11]	A, B, C, D, and E	**N/A**	ANN	89%
**Proposed method**	**A, C, and E**	**A, C, and E**	**Ensemble NSC**	**90%**
